# Doctors, Ancient and Modern

**Published:** 1881-10

**Authors:** Ausburn Towner


### DOCTORS, ANCIENT AND MODERN.

AUSBURN TOWNER.

### I.

There are few who do not know that the term doctor, like many another of our English words, has been twisted around from its original and proper signification to something entirely different therefrom. It is a Latin word and means teacher. How in our modern ways of speech it has come to be generally accepted as the title of one who practices medicine, or has sunk so much lower than that, to designate adulteration, as when it is said that the food of a man or a horse was "doctored" for some improper purpose, would be an investigation worthy the most patient etymologist. We can only suggest that some of the early teachers were bad men and put their learning to uses that so degraded their title that the bad odor has clung to it until now. Doctors, or healers, or physicians, have had their places among mankind ever since there has been a mankind, and we can judge something of the positions they have held by what we know of the savage races of the earth. Among the North American Indians, the "medicine man" is also the priest; wise in the use of herbs and roots for the healing of the sick. So it has come down to us from that ancient civilization in Egypt, of which we are learning more and more every day—that among that people the priest of their gods was also the physician. The care of the souls and the bodies is committed to the same persons. In all of those nations that presumably borrowed their civilization from the Egyptians, like the Jews, the Persians and the East Indians, these two professions, as they might be called, have never been entirely divorced. But other nations and races, like ours, the Germans and French, who get their civilization from the Greeks and Romans, filtered through the Arabians, have made of the priest and doctor two distinct persons. Even with us, however, the name doctor has been applied by compliment to clergymen as well as physicians, and in recent times also to other learned or notable men, and no one will dispute the proposition that of all professions, the clerical and the medical stand nearest together. The clerical in importance and influence being at the head of all the professions, the medical next to it in all respects, and both of them far above any other toward which the intellect and study of man can be directed.

It is only guess-work as to how much the ancient Egyptian doctors knew of the practice of medicine. Their skill in embalming would lead to the supposition that they must have been well acquainted with anatomy. From the writings of Moses also, who was learned in all the mysteries of the Egyptian priesthood, we can conclude that they knew much of the nature of disease, whether or not they could control it.

The first doctor with whom we really become acquainted in the past, is the Greek Hippocrates, who is called the "Father of Medicine," and who was born nearly 500 years before the christian era. There were other doctors before him in Greece of course, for it is related of him that he studied with them, and besides, one of his ancestors was Æsculapius, if there ever was such a person, who was said to have been the son of Apollo and was the Greek god of medicine. If Æsculapius had lived in these times and there had been said of him now what was said of him then, he would have been called a humbug. Indeed notices of his doings as they appear in classical dictionaries and encyclopedias would do no great discredit to a patent medicine advertisement of 1881, A. D. He is said to have raised men from the dead which so alarmed Jupiter for fear that the kingdom of Pluto would be depopulated, that he struck him dead with a thunderbolt. If Jupiter could come around with a few of his thunderbolts nowa-days, he might do a service to the world in many a case of a similar nature for an opposite cause.

Hippocrates was a close observer and a patient investigator. There were several of him. That is there were several physicians of that name who were conspicuous about that time. There must have been, for what are known as the experiments and observations of Hippocrates extended over a period of more than one hundred years, and a vast number of books were written bearing his name as that of their author. He knew nothing of anatomy. Before his time, the practice of medicine was largely guess-work; speculative is the polite word. He substituted experiment and observation with much success. He reduced what had been chaos to some system. He might be called the Greek Hahnemann. He discovered the critical days in fever, and was the first to proclaim the necessity and benefits of diet. His influence is still felt throughout the world in the practice of medicine. Twenty-five hundred years is a long time to be remembered.

It was not until two hundred years after Hippocrates or about 300 B. C., that any accurate human anatomical knowledge was obtained. Up to that time what was known, or supposed to be known, was gathered or guessed by analogy from the dissection of inferior animals. At this time the most distinguished doctor was Erasistratus, who was a Greek, but who practiced and taught in Alexandria. His rise to prominence was quite a romance, but he was a genius who only needed a stepping stone to greatness.

Seleucus surnamed Nicator, was one of the generals of Alexander the Great, and when that monarch died he became Satrap of Babylonia. He founded the famous Seleucidæ dynasty. His second wife was Stratonica, known as the beautiful queen of Syria. By his first wife, he had a son named Antiochus, who became Antiochus I. Now Antiochus fell into a lingering disease and none of the court physicians could tell what was the matter with him. He knew very well himself, but refused to disclose his secret. Dr. Erasistratus, in the course of his travels came into Syria and to the court of Seleucus. He was young, ambitious and not ill-looking. Antiochus was the heir apparent to the throne, and of course his health was a matter of much moment to the state. Dr. Erasistratus was shrewd or fortunate enough to discover the trouble. He told King Seleucus that when the queen Stratonica appeared in the room where the young man was, his pulse became singularly irregular, and all that ailed the prince was, he was in love with his step-mother! King Seleucus laughed, and as he had founded all his hopes on his son, and as kings in those days did pretty much as they chose with women, he gave his queen to the young prince to be his wife. The young man speedily got well and he and his beautiful queen had a long and prosperous reign. It is very certain that Dr. Erasistratus was not forgotten for his happy discovery. Antiochus heaped great wealth and favors upon him, made his name famous over the whole world, and enabled him to settle in Alexandria, where he could pursue his anatomical researches, and have no care whether he had patients or not.

The world is greatly indebted to Erasistratus although he formed some very queer notions. He came very near discovering the circulation of the blood, something that was not definitely determined until nineteen hundred years later. He however, did discover the functions of the brain, and the nervous system, and distinguished and gave names that are still used, to the auricles of the heart. His greatest discovery, however, was that of the *viæ lacteæ.* He was something of a hydropath too, as, instead of blood-letting and cathartics, he was greatly in favor of bathing, dieting and exercise. One of his singular notions which approached near to the circulation of the blood, and which, in the light of our modern knowledge sounds indeed peculiar, was, that when we breathe, we inhale that which makes life, called by him *pneuma*, or spirit. It fills the arteries and becomes the vital principle of the human system. So long as this spirit moves about in the arteries and the blood in the veins, man enjoys health; but when from any cause, the veins become contracted, the blood then gets into the arteries and becomes the source of maladies. It produces fever when it enters into some noble part or into some great artery, and inflammations, when it is found in the less noble parts or in the extremities of the arteries. We have no doubt but what Erasistratus was able to sustain this theory by some reasons that seemed incontrovertible to him, and indeed, it is not much further out of the way than many another theory that has had numerous adherents and been the foundation of an important school.

Herophilus was a brother doctor with Erasistratus, and they worked much together in Alexandria, twenty-two hundred years ago, exploring the then unknown field of anatomy. He is said to have dissected seven hundred subjects, and must have been a very busy man. He and his companion Erasistratus were exceedingly curious men too. It is said, but not on very good authority, that they opened the bodies of living criminals to discover the secret springs of life! In this respect, knowledge has not advanced one step since their time. To Herophilus belongs the credit of having first thought of that, which it seems to us should have occurred to any one long before his time, the opening of the bodies of men after death, in order to ascertain the nature of the malady that caused their dissolution. He was the first one so far as it is known, to make an autopsy or post-mortem examination for a scientific purpose, and has been called "the evangelist of anatomists." His discoveries were numerous and many of the terms that he applied to portions of the human body are in use now, twenty-two hundred years after he lived.

When we reflect upon it, it seems a long time that men have been informed accurately upon some of the portions of the human body and their functions. Luke, the physician must have been acquainted with them for these we have seen were discovered three hundred years before his time. We are not used to associate such matters together and they thus put on a peculiar appearance to us.

